# Urban vibrancy: An analogy of biodiversity, retail diversity, and activity-based urban diversity measures

**DOI:** 10.1093/pnasnexus/pgag130

**Published:** 2026-05-19

**Authors:** Edward Chung Yim Yiu, Diogo Pacheco, Riccardo Di Clemente, Federico Botta

**Affiliations:** Department of Property, University of Auckland Business School, Auckland 1010, New Zealand; Department of Computer Science, University of Exeter, Exeter EX4 4QF, United Kingdom; Complex Connections Lab, Network Science Institute, Northeastern University London, London E1W 1LP, United Kingdom; ISI Foundation, Turin 10126, Italy; Department of Computer Science, University of Exeter, Exeter EX4 4QF, United Kingdom

**Keywords:** urban vibrancy, ecological analogy, biodiversity, urban density, urban planning

## Abstract

A central question in urban studies is what makes a city vibrant—where vibrancy reflects the dynamic social energy of urban life. Traditional measures such as population density and land-use mix often fail to capture this dynamism. Drawing an analogy from ecology and retail studies, where biodiversity and retail diversity are quantified through species or tenant richness and abundance, this paper proposes a new framework for understanding urban vibrancy through the lens of activity diversity. We present a theoretical model of the key determinants of vibrancy, showing how a diversity of urban activities enhances the vitality of cities by offering a range of services and experiences without leading to overcrowding. We further extend the ecological analogy to the management of urban overdiversity, applying principles such as carrying capacity, density-dependent regulation, and niche differentiation. The paper also introduces a taxonomy for classifying urban activities in relation to vibrancy and concludes with a roadmap outlining the challenges and opportunities of this framework. Together, these insights advance our understanding of how cities thrive and offer guidance for planning strategies that balance vibrancy with sustainability.

## Introduction

The rise of urbanization in countries across the globe, coupled with the rapid increase in the availability of large digital datasets about cities, infrastructure, and human behavior, has resulted in a large number of interdisciplinary studies aiming to better understand urban environments and the interactions with people living there (1–6).

A key concept of interest is urban vibrancy, also known as urban vitality. Generally, urban vibrancy describes the energetic and dynamic activity within an urban environment. Urban vibrancy has been extensively studied in urban planning and geography, often measured through metrics, such as population density, land-use mix, and pedestrian activity (7). Jacobs (8) argued that diverse land uses contribute to vibrant urban environments by facilitating social interactions and economic exchange. More recent studies have expanded on this by incorporating spatial-temporal dynamics, showing that urban vibrancy is not solely determined by land use but also by activity patterns (9, 10). Traditionally, measuring urban vibrancy has been challenging due to the lack of data both on where people are in a city during the day and data on the dynamic structure of cities themselves. However, traditional metrics, such as population density, land-use mix, and infrastructure development, have provided valuable baseline insight for studying urban vibrancy. Yet these indicators often fail to capture the full complexity and temporal dynamism of urban life, overlooking key elements like the variety of activities, experiences, and interactions contributing to a vibrant urban environment.

In more recent years, researchers have increasingly turned to mobile data to study urban vibrancy. Researchers have used data from social media check-ins, bank transactions, public Wi-Fi usage, mobile phone records, smartphone apps, among others, to use as proxies for the presence of people and activity levels (6, 11–25) Huang et al. 2020. The notion of Active Population has recently been proposed as a better method of estimating urban interactions (26). Active Population refers to a mixture of residential and working populations, which encompasses the number of people who ever visited a certain location. These data sources and metrics enable researchers to trace real-time human movement and activity patterns, offering a more fine-grained view of how urban vibrancy changes over time and space, and its dependency on urban design and place-making. Nevertheless, they still overlook the diversity and experiential quality of activities taking place. This highlights the need for a more comprehensive framework that integrates both the quantity and variety of urban activities to better understand and plan for sustainable vibrancy. Despite these advances, there have been very few previous studies on urban activity diversity and its relationship with urban vibrancy. A study by Collins et al. (27) is one of the first attempts to observe urban activities using mobile app usage data. Yet it is limited to only seven activities and based on one source of information.

To address these gaps, this paper draws on analogies between urban environments and ecosystems, particularly those found in ecological vibrancy studies and biodiversity. In ecology, species richness and species abundance are critical metrics for understanding the vitality of an ecosystem, accounting for both the number and distribution of species (28). Similarly, in urban studies, activity diversity and abundance, which capture the variety and density of activities within a space, are essential for assessing vibrancy. This analogy has been applied to understand the effects of retail diversity on shopping mall performance in the United Kingdom (29). Retail diversity, in particular, plays a crucial role in enhancing urban vibrancy by offering a wide array of services and experiences, thereby contributing to the overall dynamism of a city.

Building on these ecological perspectives that have provided valuable insights for understanding urban vibrancy through the lens of biodiversity. In ecology, species richness and species abundance have long been used to assess the health and resilience of ecosystems (30, 31). Urban studies have drawn on these ecological principles, with some scholars suggesting that the diversity of human activities in cities mirrors the biodiversity of natural ecosystems (32, 33). However, existing urban studies have often relied on static land-use classifications rather than examining real-time activity-based diversity. By framing urban vibrancy as a form of ecological vibrancy, we propose a more comprehensive framework that incorporates activity diversity and purpose. This framework enables a deeper understanding of how urban environments function and thrive, much like how ecosystems sustain balance and vitality in their habitats.

It is widely accepted that a place lacks vibrancy if it supports only a limited range or low diversity of activities (9, 34).^1,2^ However, just as ecosystems can suffer from the negative impacts of high species density and overdiversity, such as resource depletion, species overcrowding, and the disruption caused by invasive species (35, 36), urban spaces are also vulnerable to congestion, overcrowding, and conflicts. When the variety and density of activities exceed the carrying capacity of the environment or lead to conflicting interactions between activities, urban vibrancy can also be degraded. While previous studies have generally assumed that higher population density or higher amenity density correlates with greater vibrancy, they often neglect the actual activity diversity, the issue of overdensity, and conflicts between activities (37). Similarly, urban overcrowding can result in congestion, environmental degradation, and diminished quality of life (38, 39). Ecological studies on carrying capacity and density-dependent factors provide useful parallels for understanding urban vibrancy without triggering too many negative externalities. Recent urban studies have highlighted the need for adaptive urban designs that distribute activities efficiently while maintaining vibrancy (40, 41). Thus, applying ecological principles like carrying capacity, density-dependent regulation, and niche differentiation becomes crucial in understanding urban overdiversity in land uses and activities and ensuring the long-term well-being of users of urban spaces.

There is a gap in research that explicitly models urban vibrancy using urban activity diversity metrics while addressing the risks of overcrowding and conflicts. This paper proposes a novel framework that integrates activity-based measures of urban vibrancy with ecological principles of carrying capacity and density regulation. We also suggest that studies in urban vibrancy need to move beyond simple density measures (42), such as the density of people or amenities, and instead consider a more nuanced picture of what activities people are doing or what amenities they are using and for what purpose. A recent study has also shown a fascinating switching effect in interaction patterns in cities between an active state and a sleeping state, with few large communities where people interact in the active state as opposed to many smaller communities in the sleeping state (43). It would be intriguing to explore the link between this phenomenon and vibrancy, investigating whether vibrant environments also exhibit this switching behavior between few large vibrant areas and a larger number of smaller vibrant places or uncovering whether vibrancy can only occur when cities are in their active state. Finally, we posit that social mixing is also an important determinant of urban vibrancy, and existing studies have demonstrated how social mixing can be an attractive feature of an urban space (44). Incorporating these ecological analogies provides an innovative perspective for urban planners and policymakers. By adopting an activity-based diversity framework, urban strategies can be more effectively aligned with principles of sustainability and vibrancy. This approach offers a deeper understanding of urban life, addressing the limitations of traditional metrics, and fostering the development of cities that are not only vibrant but also resilient and sustainable.

## Theoretical framework and analogies

### Determinants of urban vibrancy

To systematically assess urban vibrancy, we propose five major determinants and compare ecological vibrancy, retail vibrancy, and urban vibrancy, as can be seen in Table 1.

**Table 1:** Comparison of determinants of vibrancy in ecology, retail, and urban.

Determinant	Ecological vibrancy	Retail vibrancy	Urban vibrancy
Location/environment	Climate, habitat conditions, and resource availability	Accessibility, catchment area, and foot traffic	Accessibility, space availability, demographics, and weather
Structure/design	Ecosystem structure, habitat connectivity	Mall layout, store positioning, and pedestrian flow	Amenities, space design, synergy of private, common, and public spaces
Maintenance/management	Predator–prey balance, species interactions, ecosystem maintenance	Mall management, leasing policies, and tenant mix optimization	Cleanliness, security, order, crowd control, balance between freedom and safety
Diversity	Biodiversity, species richness, and abundance	Retail diversity, tenant mix, product variety, and shopping experience	Urban activity diversity, land-use mix, and variety of human activities
System/institution	Natural selection, ecological adaptation, and environmental policies	Market regulations, economic policies, and branding strategies	Political system, culture, inclusivity, and land rents

### Urban activity diversity and vibrancy

In ecology, species richness and species abundance measure biodiversity. A balanced ecosystem maintains a sustainable number of species through environmental factors, habitat conditions, and predator–prey dynamics. Similarly, urban areas thrive when a diverse range of activities occurs within their carrying capacities.

Retail diversity has been extensively studied concerning tenant mix, consumer preferences, and shopping mall performance (45). Just as ecosystem stability depends on species variety, shopping malls and retail districts rely on a mix of tenants to attract consumers and maintain long-term economic sustainability.

Here, we suggest that urban vibrancy emerges from a balanced mix of activities, spaces, and institutional settings. A diversified urban environment supports economic vitality, social interaction, and cultural engagement. However, excessive diversification can lead to congestion and inefficiencies, requiring urban management strategies to balance diversity with livability.

However, urban activities can be defined through two distinct approaches, each offering different, and perhaps contrasting, insights into how cities function. The first approach categorizes activities based on the designated land uses of amenities, such as residential, commercial, or recreational zones, providing a straightforward and spatially mappable understanding of urban functions. This method relies on zoning plans and amenity types to infer the activities expected to occur in those spaces. However, knowing that a place is “commercial” or “residential” does not capture what activities take place there or when and why people go there to do those activities. Thus, a second, more observational approach focuses on the actual behavior and movements of people, recognizing that individuals often engage in activities that diverge from the intended use of spaces, such as exercising in a car park or working in a café. This discrepancy poses a critical and thought-provoking challenge, both for urban planners, who are faced with decisions on how to design urban spaces that can meet both function and use, as well as for data scientists, who need to develop sophisticated tools and methodologies to capture real-time, context-sensitive human activity beyond static land-use definitions. From an urban planning perspective, the challenge is whether function or use should be prioritized and how the two could be merged in the urban spaces of the future, without distorting the feel of our cities. On the other hand, the availability of the right kind of data is a crucial question for data scientists, since the detection of individual-level activity requires suitable datasets that go beyond what is traditionally available.

### Comparison of ecological and urban vibrancy

**Figure 1:**
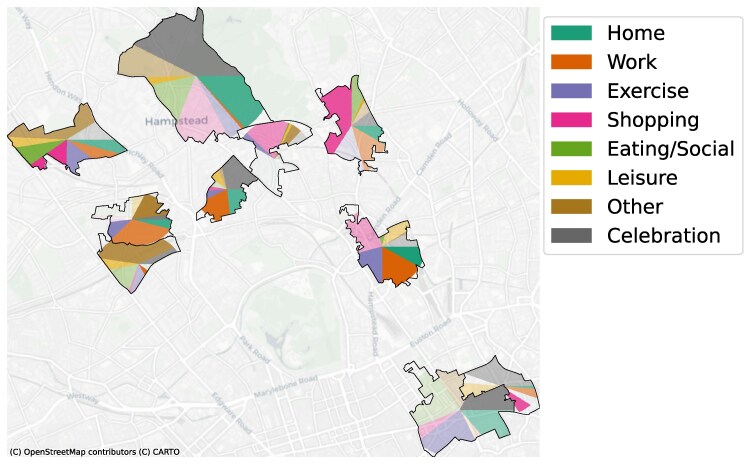
Urban vibrancy frequency and intensity. We propose that urban vibrancy should be studied using an analogy with ecology and that both the frequency (how often and how many activities) and intensity (how many people) of urban activities should be considered. Here, we depict an illustrative example of different neighborhoods in London, where each neighborhood displays a different split between different activities (in terms of how frequently they happen, as visualized by the size of the color for each activity) and intensity (how many people take part, visualized as different transparency levels). Data are for illustrative purposes only.

Table 2 outlines the key similarities and differences between biodiversity measures and urban vibrancy measures. Figure 1 presents an illustrative example of how we suggest urban environments should be studied in terms of urban vibrancy. There are different activities taking place in each neighborhood, and they can vary both in frequency (how often they take place in that environment, represented by the size of the corresponding activity) and intensity (how many people are engaging in that activity, represented by the transparency level of each activity).

**Table 2:** Comparison of biodiversity and urban diversity.

Aspect	Ecological vibrancy	Urban vibrancy
Richness	Species richness: number of different species present	Activity richness: variety of activities present
Abundance	Species abundance: population of each species	Activity abundance: frequency/number and intensity of activities/usage of amenities
Impact factors	Climate, habitat conditions, and food availability	Infrastructure, accessibility, and cultural and economic factors
Overcrowding risks	Population exceeding carrying capacity leads to competition and resource depletion	Overconcentration and conflicts of activities may reduce urban livability and cause congestion

We also further suggest that our ecological analogy can be expanded to the usage of urban spaces and the reasons for people to spend time there. A diverse range of usage of urban spaces, with different purposes by different people, can promote urban vibrancy, with the coexistence of many different human activities resembling a mutualistic association between different species (46) enriching each other's urban experiences and promoting social mixing.

### High-level categorization of urban activities

AI-driven technologies, particularly computer vision, sensor networks, and deep learning algorithms, have revolutionized how human activities are categorized and analyzed. Researchers are now able to detect and classify very specific human actions, such as standing up, sitting down, walking, running, or even more subtle movements like fidgeting or adjusting posture (47, 48). These micro-level activities are being captured through AI-powered systems, such as surveillance cameras, wearable sensors, and smartphones, enabling real-time data collection on human behavior.

For example, using AI models like convolutional neural networks, activity recognition systems can distinguish between sitting, standing, walking, or running in a public space. These models are trained to classify activities based on body posture, movement patterns, and even gestures. However, there have been very few AI models for urban activity categorization. The application of reinforcement learning algorithms can also help classify activities in urban spaces based on context, such as differentiating between “resting” and “socializing” depending on the location, time of day, and surrounding activities.

**Figure 2:**
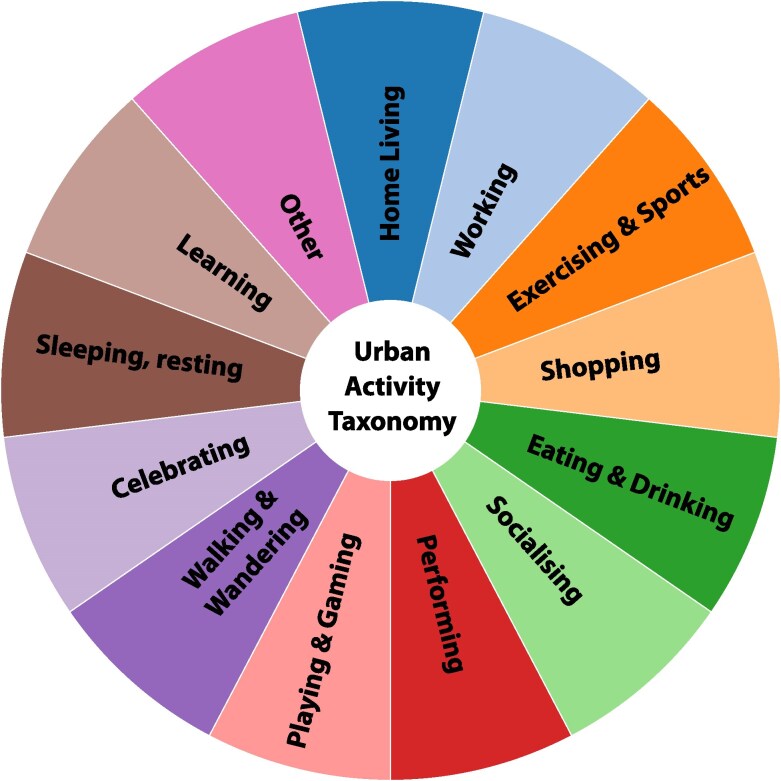
Urban activity taxonomy. We propose that urban activities can be categorized into these categories for the study of urban vibrancy.

For the purpose of studying urban vibrancy, we propose an urban taxonomy where urban activities should be categorized into the following 12 activities (Fig. 2): (i) home living, (ii) working, (iii) exercising and sports, (iv) shopping, (v) eating and drinking, (vi) networking and socializing, (vii) exhibiting, performance, and busking, (viii) playing and gaming, (ix) walking and whiling, (x) celebrating and parading, (xi) sleeping and sunbathing, (xii) learning and training, and (xiii) other activities, such as visiting museums, theaters, attending church, watching sporting events, etc. We suggest that these broad activity categories serve as a framework for understanding urban vibrancy, although detecting such granular activities will be challenging in a purely data-driven way. It is important to emphasize that our taxonomy of activities is purely based on behavior and purpose in the usage of a space, rather than on the static, designated use of a space, so that “Working” could take place in a café or park.

## Urban vibrancy data

The recent explosion in the availability of large datasets documenting our behavior and mobility, as derived from interactions with socio-technical systems, is a remarkable source of information that can be used to study urban vibrancy, among many other applications. At a high level, there are three main sources of data that are relevant to the study of urban vibrancy.

Behavioral activity data derived from interactions with mobile phones, the Internet, smartphone applications, and social media platforms can contain granular information on the location of individuals at different points in time, as well as other information, such as demographics. Data from mobile phone interactions or social media platforms can give us insight into social relationships; text-based social media posts and online reviews can provide insight into the perception of different urban places; photo-based applications provide a unique perspective into people's view of the city; and, finally, usage of different smartphone applications can shed light on the use and function of different areas. These data not only inform us about the activity density (crowdedness) and activity diversity (richness) in urban spaces, but they also identify the activity frequency and intensity (abundance).

Next, urban data from online or official providers enables researchers to relate the behavioral activity data to the urban form. Geospatial data from mapping services contains detailed information on the location of buildings and amenities, providing a rich landscape of points of interest and their locations across a city. The road network structure can be used to study accessibility and connectivity, alongside information on public transport. Recent studies have shown increasing interest in the notion of “third places,” which are all those places for spontaneous and organized social interactions, and can be identified using urban geospatial data (20, 21, 49–51). Alongside the geographical structure of urban environments, the increasing availability of urban imagery, for instance, from street-view-like images, can be used to go beyond the physical aspect of a city and study the look and feel of different urban places. It allows us to compare not only how different neighborhoods are in terms of their structure, but also how different neighborhoods with the same structure differ in terms of their visual aspect. This is something that has not been possible until recent years and remains a largely underexplored research area.

Finally, contextual data on the socioeconomic situation of cities and people living in them can help provide additional information. Census data provide information on city size, personal income, and education levels, which is likely to be correlated with people's choices of what to do and where. Crime is undoubtedly an important aspect of urban life; crime patterns and levels are likely to affect urban vibrancy. Further, secondary administrative data are often available on a city-by-city basis and can offer useful local knowledge on the impact factors of urban vibrancy.

We suggest that a combination of datasets from all the above sources be analyzed using modern deep learning, AI, and data science techniques to detect human activities in urban places, characterize activity diversity, and study urban vibrancy.

## Roadmap and challenges

In order to achieve our vision of a new framework for studying urban vibrancy, we propose a conceptual roadmap for researchers in the field. The first step is to operationalize a version of biodiversity metrics that can be adapted for urban activity measurement. Some metrics, such as richness and abundance, may be simpler to adapt to the urban context by simply considering the entropy of activities as a measure of richness, or the frequency as a measure of abundance; other metrics, however, are less trivial to adapt. For instance, carrying capacity is nontrivial in an urban context. It could refer to the maximum number of people that a specific urban environment can sustain before overcrowding becomes an issue, making people not want to spend time there anymore. Human activities could also have a carrying capacity, above which the environment may simply lose its functionality and become too chaotic to be vibrant. Identifying whether such a carrying capacity threshold exists would be of interest in the study of urban vibrancy. Touristification is a good example of a possible carrying capacity limit, where a large number of tourists flood an area and push away local residents, an effect which has been shown to also affect retail diversity (52) and which we suggest will also impact local vibrancy of an area. Amenities and businesses will also be subject to carrying capacity constraints, not only due to the amount of physical space available but also in relationship to the number of people in the area; for instance, an area with too many cafes and not enough people may lose vibrancy because people are too dispersed rather than concentrated.

The identification of human activities in the categories of our taxonomy proposed above (see Fig. 2) requires access to a range of data sources, such as smartphone apps, Wi-Fi, cameras, and other sensors, as well as significant methodological advancements in the classification and quantification of those activities. Existing work has studied how to use mobile phone data to detect transport modes (see 25, for a review), but our framework requires the ability to identify finer activities that are unlikely to be identifiable purely from mobile phone data. A hierarchical approach to identify activities in our taxonomy may be preferable initially, as data and algorithms may not be able to differentiate between some of those activities.

The temporal evolution in the activities and behaviors in different urban spaces is an important aspect to consider when thinking about the evolution of cities and their amenities. In line with what was discussed above, the mutualistic association between different behavioral activities of humans in an urban space is bound to change over time, and we envisage this dynamism to also be an important aspect of urban vibrancy. In simpler words, an environment may remain vibrant over time not despite but thanks to the dynamic changes in how people use it and why people choose to spend time there. However, the temporal dimension of urban vibrancy—how activities and spaces evolve over time—is equally crucial to understanding what makes cities vibrant.

The validation of all results is also crucial. Initially, pilots and simple experiments may be needed to validate the accuracy of new algorithms and datasets to classify activities. Then, validating the new framework across a range of cities and cultural settings to assess whether it can accurately capture vibrancy will need a series of pilots in cities across the world, as well as benchmarking with more traditional metrics. We envisage that cross-country comparisons will highlight how human behavior and urban environments are interdependent, providing supporting evidence for our analogy with biodiversity and ecological studies.

### Beyond methodological considerations, ethical implications must be addressed

Though we believe our proposal to be an exciting advancement of urban vibrancy studies, we also want to highlight that classifying and identifying human activities from digital data could be seen as a form of surveillance, which raises significant concerns about privacy and human rights. As such, ethical reviews of research projects and case studies will be crucial to ensure responsible research and innovation. The analysis of images as well as that of sensitive location (Global Positioning System) data requires careful consideration. Although common providers of street-view images tend to obfuscate or blur faces and other personal information (such as number plates), there is no guarantee that this is successful in all cases. The sharing and use of image embeddings generated by deep learning or foundation models, rather than the actual raw images, may offer a reasonable compromise in this area (54). On the other hand, privacy considerations on the use of mobile phone data and the inherently associated location information are part of a recently developed area of research, which aims to both understand privacy and deanonymization risks in mobile phone data as well as propose a model for “conscientious use” of such datasets (55–57). Recent suggestions include the creation of standardized formats for human mobility datasets (58, 59), which could help establish privacy-preserving best practices across the field. Beyond privacy concerns, the use of digital data sources, possibly coupled with AI algorithms, can introduce further challenges of inclusivity and representativeness in the data and outcomes. Digital data may not capture the experiences of all urban populations equally, potentially marginalizing communities with less access to smartphones or those who use urban spaces in ways not easily tracked by digital sensors. Here, we anticipate that careful validation, alongside ethnographic studies of people's lived experiences of urban environments, will be a key in ensuring fairness and comprehensiveness in our understanding of urban vibrancy.

## Conclusion

In this perspective paper, we have provided a brief overview of urban vibrancy research and proposed that further insights into this area can be gained by an analogy with biodiversity. Urban environments and humans interact to give rise to vibrant environments under the right conditions, where diverse human activities coexist with diverse amenities and urban features. This moves beyond the traditional dual vision of urban vibrancy with human activity and the urban fabric on different sides of the equation toward a unified framework where urban vibrancy can be better understood as a function of both the richness and abundance of human activities, balanced by spatial and infrastructural carrying capacities.

We urge a wide range of researchers from several disciplines, from urban research to computer science, ecology, policy studies, geography, and many others, to adopt this framework and advance this area of research. We also envisage urban planners, local, and national authorities, engaging with researchers in this to provide case studies and benefit from the novel understanding this will give us.

In the face of ever-increasing urbanization, designing more sustainable and more equitable cities where people can live healthier and happier lives is of paramount importance, and we envision our approach to be a significant step in that direction.

## Footnotes

1 Whyte’s (51) cases studies of urban spaces found “the basic elements of a center city – … high pedestrian volumes, and concentration and mixture of activities …” (p. 23).

2 Gehl’s (20) studies showed that “… staying activities and thereby length are the key factor for establishing lively city space and cities.” (p. 147).
